# Cytokine gene polymorphisms and serum cytokine levels in patients with idiopathic pulmonary fibrosis

**DOI:** 10.1186/1471-2350-14-66

**Published:** 2013-07-01

**Authors:** Esam H Alhamad, Joseph G Cal, Zahid Shakoor, Adel Almogren, Ahmad A AlBoukai

**Affiliations:** 1 Departments of Medicine, King Saud University, Riyadh, Saudi Arabia; 2 Departments of Pathology, King Saud University, Riyadh, Saudi Arabia; 3 Departments of Radiology, College of Medicine, King Saud University, Riyadh, Saudi Arabia; 4 Pulmonary Division, Department of Medicine (38), College of Medicine, King Saud University, P.O. Box 2925, Riyadh 11461, Saudi Arabia

**Keywords:** Idiopathic Pulmonary Fibrosis, Polymorphisms, Genotype, Cytokine

## Abstract

**Background:**

Studies have demonstrated associations between cytokine gene polymorphisms and the risk of idiopathic pulmonary fibrosis (IPF). We therefore examined polymorphisms in the genes encoding interleukin (IL)-6, IL-10, interferon gamma (IFN-γ), tumor necrosis factor alpha (TNF-α), and transforming growth factor-beta 1 (TGF-β_1_), and compared the serum levels of these cytokines in IPF patients and healthy controls. Furthermore, we examined the association of the studied genotypes and serum cytokine levels with physiological parameters and the extent of parenchymal involvement determined by high-resolution computed tomography (HRCT).

**Methods:**

Sixty patients with IPF and 150 healthy controls were included. Cytokine genotyping was performed using the polymerase chain reaction sequence specific primer (PCR-SSP) method. In a subset of patients and controls, serum cytokine levels were determined by enzyme-linked immunosorbent assay.

**Results:**

There was no difference between IPF patients and controls in the genotype and allele distributions of polymorphisms in TNF-α, IFN-γ, IL-6, IL-10, and TGF-β_1_ (all p > 0.05). The TNF-α (−308) GG, IL-6 (−174) GG and CG, and IL-10 (−1082, -819, -592) ACC ATA genotypes were significantly associated with HRCT scores (all p < 0.05). IL-10 (−1082, -819, -592) ACC haplotype was associated with the diffusion capacity of the lung for carbon monoxide, and ATA haplotype was associated with the partial pressure of oxygen (PaO_2_) (all p < 0.05). The TGF-β_1_ (codons 10 and 25) TC GG, TC GC, CC GG and CC GC genotypes were significantly associated with the PaO_2_ and HRCT scores (p < 0.05). The TGF-β_1_ (codons 10 and 25) CC GG genotype (5 patients) was significantly associated with higher PaO_2_ value and less parenchymal involvement (i.e., a lower total extent score) compared to the other TGF-β_1_ genotypes (81.5 ± 11.8 mm Hg vs. 67.4 ± 11.1 mm Hg, p = 0.009 and 5.60 ± 1.3 vs. 8.51 ± 2.9, p = 0.037, respectively). Significant differences were noted between patients (n = 38) and controls (n = 36) in the serum levels of IL-6 and IL-10 (both, p < 0.0001), but not in the levels of TNF-α and TGF-β_1_ (both, p > 0.05).

**Conclusion:**

The studied genotypes and alleles do not predispose to the development of IPF but appear to play an important role in disease severity. Our results suggest that the TGF-β_1_ (codons 10 and 25) CC GG genotype could be a useful genetic marker for identifying a subset of IPF patients with a favorable prognosis; however, validation in a larger sample is required.

## Background

Idiopathic pulmonary fibrosis (IPF) is a specific form of chronic, progressive fibrosing interstitial pneumonia of unknown cause. It occurs primarily in older adults, and is associated with the histopathological and/or radiological pattern of usual interstitial pneumonia [1]. The pathogenesis of IPF is complex and remains poorly understood. The initiation of the fibrotic response may depend upon genetic factors and environmental triggers, and T helper (Th)-1 or Th-2 cell-derived cytokines may be important. More specifically, there may be an imbalance between pro- and anti-fibrotic/inflammatory cytokines and growth factors such as tumor necrosis factor-alpha (TNF-α), transforming growth factor-beta1 (TGF-β_1_), interleukin (IL)-1Ra and IL-6 [2]. The frequencies of polymorphisms in the genes encoding IL-1Ra, TNF-α, IL-4, and IL-6 have been reported to be increased in patients with sporadic IPF, and polymorphisms of IL-6 and TGF-β_1_ have been associated with disease progression [3-9].

Pulmonary function tests and high-resolution computed tomography (HRCT) are valuable tools for evaluating patients with IPF. In addition, extent of parenchymal involvement scores (as determined by HRCT) are important prognostic markers in IPF patients [10,11]. A previous study noted that poor scores for total extent of fibrosis, honeycombing, reticulation and architectural distortion (all of which reflect advanced stage fibrosis) were significantly associated with increased mortality among Saudi IPF patients [11].

Against this background, we examined polymorphisms in the genes encoding IL-6, IL-10, interferon gamma (IFN-γ), TNF-α, and TGF-β_1_ among Saudi patients newly diagnosed with IPF, and compared our results with those from healthy volunteers. The serum levels of IL-6, IL-10, TNF-α and TGF-β_1_ were also determined among IPF patients and controls. Furthermore, we examined the associations of the studied genotypes and serum cytokine levels with physiological parameters and the extent of parenchymal involvement determined by HRCT.

## Methods

### Study population

Sixty patients with IPF and 150 healthy volunteers solicited from among the hospital personnel as control subjects were included in this study, which was performed between January 2009 and May 2011 at King Khalid University Hospital, King Saud University, Riyadh, Saudi Arabia. The study was approved by the Institutional Review Board/Ethics Committee of the College of Medicine, King Saud University, Riyadh, Saudi Arabia. Written informed consent was obtained from each individual included in the study. IPF was diagnosed according to the American Thoracic Society/European Respiratory Society consensus classification [12]. Histopathological evidence of IPF was available for 24 (40%) of patients, while the remaining patients were diagnosed on the basis of compatible clinical, laboratory and HRCT findings. Healthy controls were randomly selected and had no associated medical illness. All subjects were evaluated as outpatients, and none had clinical evidence of concurrent infection. None of the patients was receiving any corticosteroids or other immunosuppressive medications at the time of blood sample collection. In addition, none of the patients had any history of acute exacerbations of IPF within three months of the serum cytokine measurements.

Thirty patients seen during the study period were excluded because although their HRCT images were consistent with usual interstitial pneumonia, they had positive autoantibodies based on serological tests. These exclusion criteria included antinuclear antibody titer > 320, rheumatoid factor titer > 60, and/or the presence of anti-cyclic citrullinated peptide, anti-Ro/SSA, anti-La/SSB, anti-double-stranded DNA (dsDNA), anti-Smith, anti-Sclero 70 (Scl-70), anti- ribonucleoprotein (RNP), and anti-histidyl-tRNA synthetase (Jo-1).

### Measurements

Pulmonary function tests (PFT Masterscreen; Jaeger, Hoechberg, Germany) were performed using standard methodologies, including spirometry, plethysmography, and measurement of the diffusion capacity of the lung for carbon monoxide (DLco) [13-15]. Arterial blood gas values (Rapid Lab 865; Bayer, Plymouth, UK) were obtained for the partial pressure of oxygen (PaO_2_), the partial pressure of carbon dioxide (PaCO_2_), and the extent of oxygen saturation (SaO_2_).

### Chest HRCT

All patients underwent CT scanning (Light Speed 16 or VCT XT; GE Medical Systems, Milwaukee, WI, USA). Full-volume scans reconstructed every 2.5 mm were obtained throughout the entire thorax. Scans were performed during suspended inspiration with patients in the supine position. Additional limited scans using 1.25-mm thin collimation at 10-mm intervals from the aortic arch level to the lung bases, with high spatial resolution reconstruction, were obtained at end-expiration with patients in the prone position. CT images were assessed for the presence and extent of parenchymal abnormalities, including ground-glass opacity, reticular opacity, honeycombing, traction bronchiectasis, emphysema, and architectural distortion. The extent of parenchymal abnormality was determined for each complete lung using a previously described 5-point scale [16] (0 = no involvement; 1 = 1-25%; 2 = 26-50%; 3 = 51-75%; and 4 = 76-100%). Each lung was scored separately and divided into three zones (upper zone, lung apex to aortic arch; middle zone, aortic arch to a position inferior to the pulmonary veins; and lower zone, from the inferior pulmonary veins to the diaphragm). A mean score for each of the six zones was calculated for each parenchymal pattern (i.e., ground-glass opacity, reticular opacity, honeycombing, traction bronchiectasis, emphysema, and architectural distortion). Total lung involvement was determined by summing the scores for each CT pattern (total extent).

### DNA extraction from peripheral blood

Peripheral blood (8 ml) was drawn and centrifuged with an acid-citrate-dextrose (ACD) anti-coagulant. DNA extraction was performed using a QIAamp DNA mini kit (Qiagen Inc., Valencia, CA, USA) in accordance with the manufacturer’s instructions. In brief, cells were lysed with lysis buffer and proteases, the DNA was ethanol precipitated, and the sample was transferred to a QIAamp column and washed twice with washing buffer and centrifugation. To increase the DNA yield, 200 μl of elution buffer was added to the column and the sample was incubated for 5 minutes at room temperature. Finally, the DNA was collected by centrifugation for 1 minute. The concentration and purity of the recovered DNA were assessed by spectrophotometry (GeneQuantII, Pharmacia Biotech, Sweden), and the sample was stored in elution buffer at −20°C until use.

### Cytokine genotyping

The investigated gene polymorphisms included TGF-β_1_ (codons 10 and 25), IL-6 (−174), IL-10 (−1082, -819 and −592), TNF-α (−308), and IFN-γ (+874). Cytokine genotyping was performed using the polymerase chain reaction sequence specific primer (PCR-SSP) method with a cytokine-genotyping tray (Micro SSP™ primer set tray; One Lambda Inc., Canoga Park, CA, USA). DNA samples were thawed at room temperature and mixed with D-mix and recombinant Taq polymerase. The mixture was dispensed to the tray and amplification was performed in a thermocycler (Perkin Elmer 9700; Perkin Elmer, Foster City, CA, USA) using the following program: denaturation at 96°C for 2 minutes, 9 cycles of 96°C for 10 seconds and 63°C for 50 seconds, and then 20 cycles of 96°C for 10 seconds, 59°C for 50 seconds and 72°C for 30 seconds. The amplified DNA products were resolved by electrophoresis and identified using a gel-documentation system (Alpha Inotech, Santa Clara, CA, USA).

### Serum cytokine assay

Due to resource limitations, blood samples were collected from the first consecutively enrolled IPF patients (n = 38) and healthy controls (n = 36). Cytokines were assessed in serum samples by quantitative sandwich immunoassays performed on a fully automated ELISA machine (ETI-Max 3000; DiasORIN S.p.A, Vercelli, Italy) using ELISA kits purchased from R&D Systems (Minneapolis, MN, USA). Estimation of each cytokine was performed in accordance with the manufacturers’ instructions. Briefly, 50 μl of assay diluent was dispensed to each well, and 200 μl of standard, control or serum sample were added as appropriate. The contents were incubated at room temperature for 2 hours, washed four times with washing buffer, and mixed with 200 μl of cytokine conjugate per well. After a further incubation for 2 hours at room temperature, the plate was washed and 200 μl of substrate was dispensed to each well. The plate was then incubated for 20 minutes at room temperature, and 50 μl of stop solution was added to each well. Optical densities were recorded and the results were expressed in pg/ml.

### Statistical analysis

Data are presented as proportions, means and standard deviations for normally distributed data, or as median (range) for non-normally distributed data. Allele and genotype frequencies were calculated by direct counting. Observed and expected frequencies were compared using the chi-square test or Fisher’s exact test to check for Hardy-Weinberg equilibrium (HWE). Differences between genotype and allele frequencies in IPF patients and controls were analyzed with the chi-square or Fisher’s exact tests. Odds ratios and 95% confidence intervals for relative risks were calculated. One-way analysis of variance (ANOVA) and the Student’s *t*-test were used to compare the means of quantitative variables (age, physiological parameters and HRCT scores) in relation to various genotypes and serum cytokines level. For nonparametric data (serum cytokine levels between patients and controls), the Mann–Whitney U test was used. Pearson’s and Spearman’s correlation coefficients were used for parametric and nonparametric data, respectively, to examine the relationship between serum cytokine levels and quantitative variables. A two-sided p value < 0.05 was considered statistically significant. All analyses were performed using the Statistical Software Package for the Social Sciences (SPSS, version 16.0; SPSS Inc., Chicago, IL, USA).

## Results

The 150 healthy controls included 75 males and 75 females with a mean age of 30.8 ± 9.6 years. Among the 60 IPF patients, the mean age was 61.1 ± 12.9 years; there was a slight predominance of males (33; 55%), and the male-to-female ratio was 1.22:1. The demographic and clinical characteristics of the IPF patients are shown in Table 1. As expected, restrictive ventilatory defects with markedly decreased diffusion capacities of the lung for carbon monoxide were commonly noted among the IPF patients.

**Table 1:** Demographic and clinical characteristics of IPF patients and healthy controls

**Characteristics**	**Patients (n = 60)**	**Healthy controls**
Age at presentation, years	61.1 ± 12.9	30.8 ± 9.6
Male/Female	33/27	75/75
Disease duration, months	32.6 ± 12.9	-
Ever smoker, n (%)	20 (33.3)	34 (22.6)
**Baseline PFTs**		
FVC,% predicted	61.7 ± 21.9	-
FEV_1_,% predicted	69.8 ± 23.8	-
TLC,% predicted	57.5 ± 18.7	-
DLco,% predicted	38.1 ± 21.3	-
Baseline ABG		
PaO_2_, mmHg	68.6 ± 11.7	-
PaCO_2_, mmHg	41.1 ± 6.0	-
SaO_2_,%	93.9 ± 3.7	-
HRCT scores		
Total extent	8.3 ± 3.0	-
Ground glass opacity	1.4 ± 0.8	-
Reticulation	1.6 ± 0.7	-
Honeycombing	1.4 ± 0.6	-
Traction Bronchiectasis	2.1 ± 1.0	-
Architectural distortion	1.2 ± 0.8	-
Emphysema	0.4 ± 0.8	-

Data are presented as means ± standard deviations or number (with percentages).

Abbreviations: *IPF* idiopathic pulmonary fibrosis, *PFTs* pulmonary function tests, *ABG* arterial blood gas, *FVC* forced vital capacity, *FEV*_*1*_ forced expiratory volume in 1 second, *TLC* total lung capacity, *DLco* diffusion capacity of lung for carbon monoxide, *PaO*_*2*_ partial pressure of oxygen, *PaCO*_*2*_ partial pressure of carbon dioxide, *SaO*_*2*_ oxygen saturation and *HRCT* high-resolution computed tomography.

The distributions of the observed genotypes were not significantly different from the expected distribution according to HWE (Table 2) (all p > 0.05).

**Table 2:** Hardy-Weinberg equilibrium tests for the investigated cytokine gene polymorphisms among the healthy controls

**Cytokine/genotype**	**Observed**	**Expected**	*p-value
TNF-α (−308)			
AA	6.0	3.5	0.274
AG	25.3	30.3	0.304
GG	68.7	66.2	0.622
IFN-γ (+874)			
TT	23.3	22.7	0.891
AT	48.7	49.9	0.817
AA	28.0	27.4	0.897
IL-6 (−174)			
CC	6.0	2.9	0.156
CG	22.0	28.2	0.230
GG	72.0	68.9	0.612
IL-10 (−1082, -819, -592)			
GCC GCC	18.7	16.0	0.542
GCC ACC	20.7	22.4	0.674
GCC ATA	22.0	25.6	0.497
ACC ACC	10.7	7.8	0.472
ACC ATA	14.0	17.9	0.345
ATA ATA	14.0	10.2	0.286
TGF-β_1_ (codons 10 and 25)			
TT GG	31.3	26.3	0.373
TC GG	35.3	37.6	0.719
TC GC	10.0	13.5	0.368
CC GG	18.0	12.3	0.199
TT GC	0	0	-
CC GC	4.7	8.8	0.164
CC CC	0.7	1.4	0.562
TT CC	0	0	-
TC CC	0	0	-

Data are presented as percentages.

Abbreviations: *TNF-α* tumor necrosis factor alpha, *IFN-γ* interferon gamma, *IL* interleukin and *TGF-β*_*1*_ transforming growth factor beta 1.

* Chi-square or Fisher’s exact tests were used to compare frequencies between observed and expected genotypes.

The distributions of the different cytokine genotypes and alleles in the IPF and healthy control groups are shown in Tables 3, 4 and 5. There was no significant difference in the genotype or allele distributions of polymorphisms in TNF-α (−308), IFN-γ (+874), IL-6 (−174), IL-10 (−1082, -819 and −592), and TGF-β_1_ (codons 10 and 25) between the IPF and healthy control groups (all p > 0.05).

**Table 3:** Comparison of genotype and allele frequencies for TNF-α, IFN-γ, and IL-6 in IPF patients and healthy controls

**Cytokine/Genotype/Allele**	**IPF**	**Healthy controls**	*p-value	**OR**	**95% CI**
	**(n = 60)**	**(n = 150)**			
TNF-α (−308)					
AA^a^	4 (6.7)	9 (6)	0.856	0.894	0.264 – 3.020
AG^b^	19 (31.7)	38 (25.3)	0.392	0.732	0.380 – 1.412
GG^c^	37 (61.7)	103 (68.7)	0.336	1.362	0.730 – 2.544
A allele^d^	27 (22.5)	56 (18.7)	0.416	0.791	0.471 – 1.327
G allele	93 (77.5)	244 (81.3)			
IFN-γ (+874)					
TT^e^	15 (25)	35 (23.3)	0.858	0.913	0.455 – 1.832
AT^f^	23 (38.3)	73 (48.7)	0.220	1.525	0.828 – 2.810
AA^g^	22 (36.7)	42 (28)	0.247	0.672	0.356 – 1.267
T allele^h^	53 (44.2)	143 (47.7)	0.588	0.868	0.567 – 1.329
A allele	67 (55.8)	157 (52.3)			
IL-6 (−174)					
CC^i^	4 (6.7)	9 (6)	0.856	0.894	0.264 – 3.020
CG^j^	11 (18.3)	33 (22)	0.708	1.256	0.588 – 2.685
GG^k^	45 (75)	108 (72)	0.733	0.857	0.432 – 1.699
C allele^l^	19 (15.8)	51 (17)	0.885	1.089	0.612 – 1.935
G allele	101 (84.2)	249 (83)			

Data are presented as number (with percentages).

Abbreviations: *TNF-α* tumor necrosis factor alpha, *IFN-γ* interferon gamma, *IL* interleukin, *IPF* idiopathic pulmonary fibrosis, *OR* odds ratio, *CI* confidence interval.

*Chi-square or Fisher’s exact tests were used to compare frequencies between genotypes and alleles.

^a^AA vs. GG and AG genotypes.

^b^AG vs. AA and GG genotypes.

^c^GG vs. AG and AA genotypes.

^d^A vs. G allele.

^e^TT vs. AT and AA genotypes.

^f^AT vs. AA and TT genotypes.

^g^AA vs. TT and AT genotypes.

^h^T vs. A allele.

^i^CC vs. GG and CG genotypes.

^j^CG vs. CC and GG genotypes.

^k^GG vs. CG and CC genotypes.

^l^C vs. G allele.

**Table 4:** Comparison of genotype, haplotype carrier rate, haplotype carrier frequencies and allele frequencies for IL-10 in IPF patients and healthy controls

**IL-10 (−1082, -819, -592)**		**IPF (n = 60)**	**Healthy controls (n = 150)**	*p-value	**OR**	**95% CI**
Genotype						
GCC GCC^a^		10 (16.7)	28 (18.7)	0.844	1.148	0.519 – 2.537
GCC ACC^b^		13 (21.7)	31 (20.7)	0.853	0.942	0.454 – 1.955
GCC ATA^c^		14 (23.3)	33 (22)	0.856	0.927	0.455 – 1.889
ACC ACC^d^		7 (11.7)	16 (10.7)	0.811	0.904	0.352 – 2.322
ACC ATA^e^		8 (13.3)	21 (14)	1.000	1.058	0.441 – 2.540
ATA ATA^f^		8 (13.3)	21 (14)	1.000	1.058	0.441 – 2.540
Haplotype carrier rate						
GCC carriers		37 (61.7)	92 (61.3)	0.964	0.986	0.533 – 1.825
ACC carriers		28 (46.7)	68 (45.3)	0.861	0.948	0.520 – 1.728
ATA carriers		30 (50)	75 (50)	1.000	1.000	0.549 – 1.820
Haplotype frequency						
GCC		0.392	0.400	0.935	1.021	0.616 – 1.693
ACC		0.292	0.280	0.737	0.916	0.549 – 1.528
ATA		0.316	0.320	0.993	0.998	0.602 – 1.653
Allele						
−1082	G allele^g^	47 (39.2)	120 (40)	0.912	0.966	0.626 – 1.489
	A allele	73 (60.8)	180 (60)			
−819	C allele^h^	82 (68.3)	204 (68)	1.000	1.015	0.644 – 1.600
	T allele	38 (31.7)	96 (32)			
−592	C allele^i^	82 (68.3)	204 (68)	1.000	1.015	0.644 – 1.600
	A allele	38 (31.7)	96 (32)			

Data are presented as number (with percentages).

Abbreviations: *IL* interleukin, *IPF* idiopathic pulmonary fibrosis, *OR* odds ratio, *CI* confidence interval.

*Chi-square or Fisher’s exact tests were used to compare frequencies between genotypes and alleles.

^a^GCC GCC vs. other IL-10 genotypes.

^b^GCC ACC vs. other IL-10 genotypes.

^c^GCC ATA vs. other IL-10 genotypes.

^d^ACC ACC vs. other IL-10 genotypes.

^e^ACC ATA vs. other IL-10 genotypes.

^f^ATA ATA vs. other IL-10 genotypes.

^g^G vs. A allele.

^h^C vs. T allele.

^i^C vs. A allele.

**Table 5:** **Comparison of genotype and allele frequencies for TGF-β**_**1 **_**in IPF patients and healthy controls**

**Cytokine/genotype/allele**		**IPF (n = 60)**	**Healthy controls (n = 150)**	*p-value	**OR**	**95% CI**
TGF-β_1_ (codons 10 and 25)						
TT GG^a^		24 (40)	47 (31.3)	0.260	0.684	0.368 – 1.274
TC GG^b^		24 (40)	53 (35.3)	0.530	0.820	0.443 – 1.517
TC GC^c^		3 (5)	15 (10)	0.289	2.111	0.588 – 7.575
CC GG^d^		5 (8.3)	27 (18)	0.091	2.415	0.883 – 6.602
TT GC		0	0	-		
CC GC^e^		3 (5)	7 (4.7)	0.918	0.930	0.232 – 3.723
CC CC^f^		1 (1.7)	1 (0.7)	0.500	0.396	0.024 – 6.435
TT CC		0	0	-		
TC CC		0	0	-		
Codon 10	T allele^g^	75 (62.5)	162 (54)	0.128	1.420	0.920 – 2.191
	C allele	45 (37.5)	138 (46)			
Codon 25	G allele^h^	112 (93.3)	276 (92)	0.839	1.217	0.531 – 2.791
	C allele	8 (6.7)	24 (8)			

Data are presented as number (with percentages).

Abbreviations: *TGF-β*_*1*_ transforming growth factor beta, *IPF* idiopathic pulmonary fibrosis, *OR* odds ratio, *CI* confidence interval;

*Chi-square or Fisher’s exact tests were used to compare frequencies between genotypes and alleles.

^a^TT GG vs. other TGF-β_1_ genotypes.

^b^TC GG vs. other TGF-β_1_ genotypes.

^c^TC GC vs. other TGF-β_1_ genotypes.

^d^CC GG vs. other TGF-β_1_ genotypes.

^e^CC GC vs. other TGF-β_1_ genotypes.

^f^CC CC vs. other TGF-β_1_ genotypes.

^g^T vs. C allele.

^h^G vs. C allele.

The associations of the studied genotypes/alleles with the physiological parameters and CT scores for the extent of parenchymal abnormalities are shown in Tables 6 and 7.

**Table 6:** Associations of TNF-α, IFN-γ, and IL-6 polymorphisms with physiological parameters and HRCT scores in IPF patients

**Cytokine/genotype/**	**†p-value**								
**Allele**	**PaO**_**2**_	**FVC, % P**	**DLco,% P**	**Total extent**	**GGO**	**Retic**	**HC**	**AD**	**Emphy**
TNF-α (−308)									
GG^a^	0.670	0.397	0.663	0.214	0.348	0.361	0.036*	0.147	0.164
AG^b^	0.563	0.937	0.845	0.407	0.371	0.670	0.052	0.107	0.333
AA^c^	0.806	0.132	0.643	0.387	0.873	0.326	0.656	0.860	0.372
ANOVA	0.839	0.303	0.859	0.415	0.638	0.512	0.108	0.273	0.346
A allele^d^	0.803	0.179	0.578	0.161	0.397	0.238	0.055	0.262	0.125
G allele									
IFN-γ (+874)									
TT^e^	0.574	0.725	0.913	0.769	0.603	0.916	0.904	0.924	0.861
AT^f^	0.923	0.552	0.572	0.736	0.211	0.875	0.938	0.543	0.860
AA^g^	0.547	0.360	0.652	0.939	0.430	0.950	0.852	0.484	0.983
ANOVA	0.791	0.659	0.842	0.933	0.460	0.987	0.982	0.763	0.979
T allele^h^	0.443	0.392	0.808	0.896	0.824	0.982	0.838	0.586	0.925
A allele									
IL-6 (−174)									
GG^i^	0.236	0.654	0.346	0.138	0.021*	0.109	0.719	0.503	0.861
CG^j^	0.213	0.530	0.332	0.325	0.022*	0.502	0.674	0.663	0.686
CC^k^	0.901	0.844	0.860	0.300	0.659	0.083	0.198	0.626	0.351
ANOVA	0.444	0.817	0.600	0.309	0.057	0.148	0.425	0.785	0.623
C allele^l^	0.288	0.777	0.379	0.078	0.032*	0.028*	0.352	0.420	0.547
G allele									

Abbreviations: *TNF-α* tumor necrosis factor alpha, *IFN-γ* interferon gamma, *IL* interleukin, *HRCT* high-resolution computed tomography, *IPF* idiopathic pulmonary fibrosis, *PaO*_*2*_ partial pressure oxygen, *FVC,% P* forced vital capacity, percent predicted, *DLco* diffusion capacity of lung for carbon monoxide, *GGO* ground glass opacity, *Retic* reticulation, *HC* honeycombing, *AD* architectural distortion, *Emphy* emphysema.

† One-way analysis of variance (ANOVA) and the Student’s *t*-test were used to compare the means of quantitative variables.

* Statistically significant.

^a^GG vs. AG and AA genotypes.

^b^AG vs. AA and GG genotypes.

^c^AA vs. GG and AG genotypes.

^d^A vs. G allele.

^e^TT vs. AT and AA genotypes.

^f^AT vs. AA and TT genotypes.

^g^AA vs. TT and AT genotypes.

^h^T vs. A allele.

^i^GG vs. CG and CC genotypes.

^j^CG vs. CC and GG genotypes.

^k^CC vs. GG and CG genotypes.

^l^C vs. G allele.

**Table 7:** **Association of IL-10 and TGF-β**_**1 **_**polymorphisms with physiological parameters and HRCT scores in IPF patients**

**Cytokine/genotype/**	**†p-value**								
**Allele**	**PaO**_**2**_	**FVC, % P**	**DLco,% P**	**Total extent**	**GGO**	**Retic**	**HC**	**AD**	**Emphy**
IL-10 (−1082, -819, -592)									
GCC GCC^a^	0.389	0.262	0.948	0.098	0.137	0.935	0.514	0.334	0.173
GCC ACC^b^	0.583	0.286	0.411	0.278	0.552	0.853	0.619	0.171	0.778
GCC ATA^c^	0.390	0.905	0.710	0.564	0.741	0.942	0.501	0.539	0.962
ACC ACC^d^	0.252	0.456	0.531	0.437	0.341	0.536	0.658	0.884	0.460
ACC ATA^e^	0.533	0.354	0.390	0.171	0.837	0.069	0.014*	0.081	0.388
ATA ATA^f^	0.319	0.730	0.058	0.695	0.837	0.147	0.203	0.820	0.635
ANOVA	0.587	0.661	0.469	0.333	0.698	0.416	0.173	0.393	0.719
GCC haplotype^g^	0.606	0.472	0.517	0.497	0.120	0.415	0.769	0.681	0.333
ACC haplotype^h^	0.312	0.293	0.030*	0.329	0.080	0.326	0.440	0.767	0.101
ATA haplotype^i^	0.018*	0.862	0.097	0.802	0.799	0.858	0.769	0.907	0.783
−1082 G allele^j^	0.461	0.499	0.830	0.438	0.144	0.876	0.996	0.881	0.200
A allele									
−819 C allele^k^	0.053	0.841	0.066	0.941	0.914	0.542	0.617	0.793	0.988
T allele									
−592 C allele^l^	0.053	0.841	0.066	0.941	0.914	0.542	0.617	0.793	0.988
A allele									
TGF-β_1_(codons 10 and 25)									
TT GG^m^	0.902	0.161	0.548	0.690	0.462	0.708	0.734	0.839	0.711
TC GG^n^	0.967	0.290	0.663	0.509	0.017*	0.708	0.734	0.892	0.951
TC GC^o^	0.041*	0.119	0.217	0.876	0.240	0.528	0.270	0.732	0.444
CC GG^p^	0.009*	0.372	0.526	0.037*	0.238	0.201	0.146	0.248	0.314
TT GC									
CC GC^q^	0.128	0.858	0.101	0.412	0.020*	0.291	0.074	0.059	0.945
CC CC^r^	0.911	0.083	0.539	0.451	0.090	0.403	0.533	0.122	0.050
TT CC									
TC CC									
ANOVA	0.025*	0.157	0.408	0.359	0.010*	0.586	0.246	0.210	0.382
Codon 10 T allele^s^	0.662	0.076	0.441	0.315	0.319	0.543	0.701	0.760	0.738
C allele									
Codon 25 G allele^t^	0.021*	0.687	0.027*	0.890	0.053	0.726	0.968	0.800	0.370
C allele									

Abbreviations: *IL* interleukin, *TGF- β*_*1*_ transforming growth factor beta, *HRCT* high-resolution computed tomography, *IPF* idiopathic pulmonary fibrosis, *PaO*_*2*_ partial pressure of oxygen, *FVC,% P* forced vital capacity, percent predicted, *DLco* diffusion capacity of the lung for carbon monoxide, *GGO* ground glass opacity, Retic reticulation, *HC* honeycombing, *AD* architectural distortion, *Emphy* emphysema.

† One-way analysis of variance (ANOVA) and the Student’s *t*-test were used to compare the means of quantitative variables.

* Statistically significant.

^a^GCC GCC vs. other IL-10 genotypes.

^b^GCC ACC vs. other IL-10 genotypes.

^c^GCC ATA vs. other IL-10 genotypes.

^d^ACC ACC vs. other IL-10 genotypes.

^e^ACC ATA vs. other IL-10 genotypes.

^f^ATA ATA vs. other IL-10 genotypes.

^g^GCC vs. other IL-10 haplotypes.

^h^ACC vs. other IL-10 haplotypes.

^i^ATA vs. other IL-10 haplotypes.

^j^G vs. A allele.

^k^C vs. T allele.

^l^C vs. A allele.

^m^TT GG vs. other TGF-β_1_ genotypes.

^n^TC GG vs. other TGF-β_1_ genotypes.

^o^TC GC vs. other TGF-β_1_ genotypes.

^p^CC GG vs. other TGF-β_1_ genotypes.

^q^CC GC vs. other TGF-β_1_ genotypes.

^r^CC CC vs. other TGF-β_1_ genotypes.

^s^T vs. C allele.

^t^G vs. C allele.

Patients with the TNF-α (−308) GG genotype had a higher mean score for honeycombing compared to those with the other TNF-α (−308) genotypes (1.51 ± 0.61 vs. 1.17 ± 0.57, respectively, p = 0.036), whereas patients with the TNF-α (−308) AG genotype tended to have a lower honeycombing extent score compared to those with the other TNF-α (−308) genotypes (1.16 ± 0.60 vs. 1.49 ± 0.59, respectively, p = 0.052). Furthermore, honeycombing extent score tended to be lower in patients with the TNF-α (−308) A allele versus the G allele (1.19 ± 0.56 vs. 1.44 ± 0.62, respectively, p = 0.055).

The mean ground glass opacity score was significantly higher for the IL-6 (−174) GG genotype versus the other IL-6 (−174) genotypes (1.58 ± 0.89 vs. 1.00 ± 0.54, respectively, p = 0.021), whereas the IL-6 (−174) CG genotype was associated with significantly lower ground glass opacity extent scores compared to the other IL-6 (−174) genotypes (0.91 ± 0.54 vs. 1.55 ± 0.87, respectively, p = 0.022). Moreover, the IL-6 (−174) C allele was significantly associated with lower ground glass opacity and reticulation extent scores versus the G allele (1.05 ± 0.52 vs. 1.50 ± 0.88, p = 0.032 and 1.26 ± 0.65 vs. 1.64 ± 0.69, p = 0.028, respectively).

Patients with the IL-10 (−1082, -819, -592) ACC ATA genotype had significantly higher honeycombing extent scores compared to those with the other IL-10 (−1082, -819, -592) genotypes (1.88 ± 0.64 vs. 1.31 ± 0.58, p = 0.014). The IL-10 (−1082, -819, -592) ACC haplotype was associated with higher DLco value compared to the other haplotypes (43.18 ± 24.11% predicted vs. 32.38 ± 17.99% predicted, p = 0.030), and the ATA haplotype was associated with a lower PaO_2_ than the other haplotypes (65.58 ± 11.14 mm Hg vs. 71.74 ± 12.12 mmHg, p = 0.018).

Exploring the association of TGF-β_1_ (codons 10 and 25) with physiological parameters and CT scores revealed several significant findings. The TGF-β_1_ (codons 10 and 25) TC GG genotype was significantly associated with a higher ground glass opacity extent score compared to the other TGF-β_1_(codons 10 and 25) genotypes (1.75 ± 0.79 vs. 1.22 ± 0.83, p = 0.017), and the TGF-β_1_ (codons 10 and 25) TC GC genotype was associated with a lower PaO_2_ than the other TGF-β_1_ (codons 10 and 25) genotypes (55.16 ± 4.13 mm Hg vs. 69.32 ± 11.59 mmHg, p = 0.041). In contrast, the TGF-β_1_ (codons 10 and 25) CC GG genotype was associated with a higher PaO_2_ and less parenchymal involvement (i.e., a lower total extent score) compared to the other TGF-β_1_ (codons 10 and 25) genotypes (81.50 ± 11.86 mm Hg vs. 67.44 ± 11.11 mm Hg, p = 0.009 and 5.60 ± 1.34 vs. 8.51 ± 2.99, p = 0.037, respectively). The CC GC genotype was associated with a lower ground glass opacity score compared to the other genotypes (0.33 ± 0.57 vs. 1.49 ± 0.83, p = 0.02), and the TGF-β_1_ (codons 10 and 25) G allele was associated with higher PaO_2_ values than the C allele (69.26 ± 11.75 mm Hg vs. 59.46 ± 5.82 mm Hg, p = 0.021).

**Figure 1:**
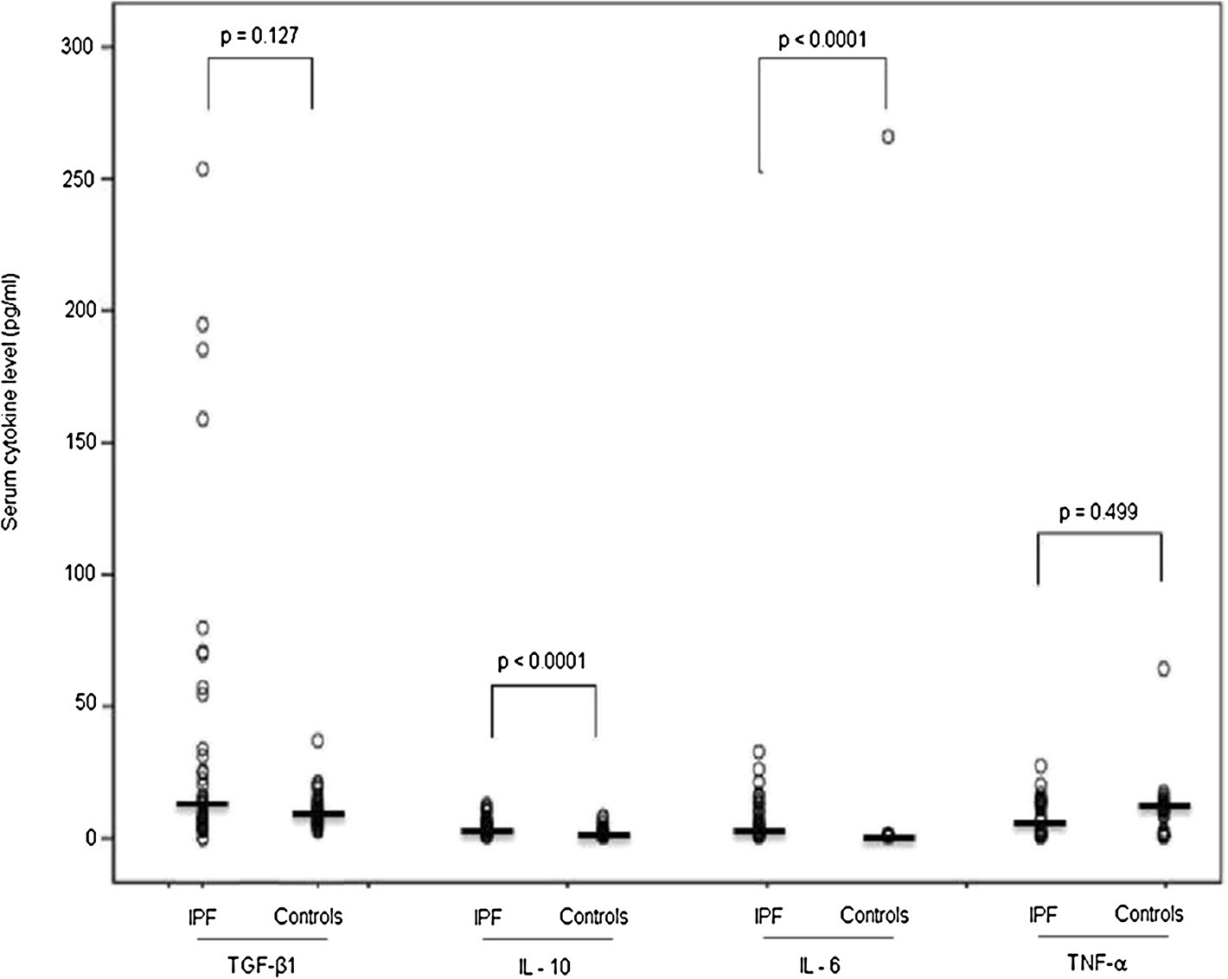
**Serum levels of interleukin (IL)-6, IL-10, transforming growth factor-beta 1 (TGF-β**_**1**_**), and tumor necrosis factor alpha (TNF-α) in idiopathic pulmonary fibrosis (IPF) patients (n = 38) and healthy controls (n = 36).** Each circle represents one individual, and transverse lines indicate median values. P-values were determined using the nonparametric Mann–Whitney U test.

Comparisons of serum cytokine levels in patients (n = 38) and controls (n = 36) are shown in Figure 1.

Among the IPF patients, the serum levels of IL-6 [median 4.03 pg/ml (range 0.00 – 32.35 pg/ml), p < 0.0001] and IL-10 [median 3.56 pg/ml (range 0.00 – 12.17 pg/ml), p < 0.0001] were significantly higher than those in the healthy controls [median 0.00 pg/ml (range 0.00 – 267.38 pg/ml) for IL-6 and median 0.8 pg/ml (range 0.00 – 7.56 pg/ml for IL-10)].

There was no significant difference in the serum levels of TNF-α between the IPF patients and controls [median 3.3 pg/ml (range 0.00 - 26.90 pg/ml and median 10.6 pg/ml (range 0.00 – 64.07 pg/ml), respectively; p = 0.499]. In addition, we found no significant difference in the serum levels of TGF-β_1_ between IPF patients and controls [median 13.7 pg/ml (range 0.00 - 253.84 pg/ml) and median 10.2 pg/ml (range 2.67 – 37.15 pg/ml), respectively; p = 0.127].

The biochemical serum characteristics of the IPF patients and healthy controls in relation to their genotypes (high, intermediate, and low producers) are shown in Table 8.

**Table 8:** Serum cytokine levels in relation to their genotypes and alleles among IPF patients and healthy controls

**Cytokine/genotype/ Allele**	**Producer**	**n**	**IPF**	**n**	**Healthy controls**	*p-value
TNF-α						
GG	Low	21	1.37 (0 – 19.68)	24	11.22 (0 – 64.07)	0.226
GA	High	13	6.82 (0 – 26.90)	12	8.64 (0 – 11.58)	0.511
AA	High	4	9.99 (1.27 – 13.10)	-		
G allele		34	2.35 (0 – 26.9)	36	10.81 (0 – 64.07)	0.194
A allele		17	7.89 (0 – 26.9)	12	8.64 (0 – 11.58)	0.293
IL-6						
GG	High	27	3.44 (0 – 32.35)	24	0 (0 – 267.38)	<0.0001
GC	High	8	6.45 (0.57 – 15.41)	8	0 (0 – 0.58)	<0.0001
CC	Low	3	14.1 (4.80 – 16.11)	4	0	0.057
G allele		35	3.61 (0 – 32.35)	32	0 (0 – 267.38)	<0.0001
C allele		11	9.52 (0.57 – 16.11)	12	0 (0 – 0.58)	<0.0001
IL-10						
GCC GCC	High	6	4.99 (0 – 11.27)	7	0.56 (0 – 2.03)	0.031
GCC ACC	Intermediate	8	3.61 (0 – 7.16)	6	1.44 (0 – 3.38)	0.090
GCC ATA	Intermediate	9	3.48 (0 – 9.48)	8	0.10 (0 – 7.56)	0.077
ACC ACC	Low	6	4.05 (0 – 6.52)	7	1.58 (0 – 4.92)	0.221
ACC ATA	Low	3	2.99 (0.72 – 3.56)	4	1.54 (0 – 6.74)	0.721
ATA ATA	Low	6	4.92 (0.80 – 12.16)	4	0.57 (0 – 5.13)	0.087
GCC haplotype		23	3.56 (0 – 11.27)	21	0.41 (0 – 7.56)	0.001
ACC haplotype		17	3.56 (0 – 7.16)	17	1.48 (0 – 6.74)	0.067
ATA haplotype		18	3.56 (0 – 11.27)	16	0.10 (0 – 7.56)	0.011
−1082 G allele		23	3.56 (0 – 11.27)	21	0.48 (0 – 7.56)	<0.0001
A allele		32	3.58 (0 – 12.16)	29	1.09 (0 – 7.56)	<0.0001
−819 C allele		32	3.56 (0 – 11.27)	32	1.22 (0 – 7.56)	<0.0001
T allele		18	4.50 (0 – 12.16)	16	0.10 (0 – 7.56)	0.002
−592 C allele		32	3.56 (0 – 11.27)	32	1.22 (0 – 7.56)	<0.0001
A allele		18	4.50 (0 – 12.16)	16	0.10 (0 – 7.56)	0.002
TGF-β_1_						
TT GG	High	14	18.98 (0 – 253.84)	13	10.34 (2.67 – 19.34)	0.058
TC GG	High	17	9.7 (0.16 – 185.28)	12	10.23 (5.27 – 21.21)	0.929
TC GC	Intermediate	3	12.95(3.44 – 70.94)	5	8.04 (3.96 – 37.15)	0.881
CC GG	Intermediate	3	6.32 (4.5 – 13.72)	5	10.58 (6.06 – 13.19)	0.655
TT GC	Intermediate		-		-	
CC GC	Low	1	16.35	1	6.91	0.317
CC CC	Low		-		-	
TT CC	Low		-		-	
TC CC	Low		-		-	
Codon 10 T allele		34	14.87 (0 – 253.84)	30	10.34 (2.67 – 37.15)	0.023
C allele		24	11.32 (0.16 – 185.28)	23	10.11 (3.96 – 37.15)	0.949
Codon 25 G allele		38	13.33 (0 – 253.84)	23	10.34 (2.67 – 37.15)	0.067
C allele		4	14.65 (3.44 – 70.94)	19	7.47 (3.96 – 37.15)	0.670

Data presented as median (range) in pg/ml. n = number of patients.

Abbreviations: *TNF-α* tumor necrosis factor alpha, *IL* interleukin, and *TGF-β*_*1*_ transforming growth factor beta.

*Nonparametric Mann–Whitney U test.

The relationship between serum levels of IL-10 and IL-10 haplotype-carrier state were examined. The serum levels of IL-10 were not significantly different among the IPF who carried the GCC haplotype [median 3.56 pg/ml (range 0.00 – 11.27 pg/ml)] compared with the levels in GCC haplotype-negative patients [median 4.50 pg/ml (range 0.00 – 12.16 pg/ml)] (p = 0.701). Furthermore, no significant difference in the serum levels of IL-10 among the IPF who carried the ACC haplotype [median 3.56 pg/ml (range 0.00 – 7.16 pg/ml)] compared with the levels in ACC haplotype-negative patients [median 4.50 pg/ml (range 0.00 – 12.16 pg/ml)] (p = 0.281). Moreover, no significant difference in the serum levels of IL-10 among the IPF who carried the ATA haplotype [median 3.56 pg/ml (range 0.00 – 12.16 pg/ml)] compared with the levels in ATA haplotype-negative patients [median 3.61 pg/ml (range 0.00 – 11.27 pg/ml)] (p = 0.988). Among the healthy controls no significant difference in serum levels of IL-10 were noted in relation to IL-10 haplotypes (data not shown).

Correlation analysis did not show any significant relationship between the studied serum cytokine levels and the physiological parameters or CT scores for the extent of parenchymal abnormalities in our IPF patients (data not shown).

## Discussion

In the present study, we observed significant associations between TNF-α, IL-6, IL-10, and TGF- β_1_ polymorphisms and PaO_2_, DLco and HRCT scores. Furthermore, the serum cytokine levels of IL-6 and IL-10 were significantly higher in IPF patients compared to healthy controls.

IPF is a disabling fibroproliferative disorder characterized by progressive fibrosis of the interstitial spaces of the lung, resulting in destruction of the normal parenchymal architecture [17]. Despite extensive research, the cause of IPF is still unknown. Substantial evidence in animal models and humans supports the hypothesis that there is an imbalance between Th-1 and Th-2 cytokines, with an excess of Th-2 cytokines being associated with the development of lung fibrosis [2,18,19].

IL-10 is a T-cell-derived cytokine of the Th-2 family that is known to suppress inflammation by inhibiting a number of pro-inflammatory cytokines [20]. Moreover, IL-10 has been shown to induce the generation of a high-IL-10-producing subset of CD4+ T cells, called regulatory T cells, that are capable of down-regulating antigen-specific immune responses [21]. The pro-fibrotic activity of IL-10 is currently under debate, as experimental studies have reported both anti-fibrotic [22] and pro-fibrotic [23] activities. Martinez and colleagues [24] noted that alveolar macrophages recovered from bronchoalveolar lavage (BAL) in patients with pulmonary fibrosis showed increased IL-10 mRNA expression. Notably, however, these patients had lower IL-10 protein levels in their BAL fluid (BALF) compared to healthy control subjects. Our study confirms the findings of Tsoutsou et al. [25] that IL-10 is markedly increased in the sera of IPF patients compared to healthy controls. The clinical significance of the high levels of IL-10 observed in IPF patients is unclear, as we found no association between serum IL-10 levels and physiological parameters or the extent of parenchymal abnormalities based on CT scores among the IPF patients. Nevertheless, we found that the IL-10 (−1082, -819, -592) ACC ATA genotype was significantly associated with a higher honeycombing extent score (i.e., a less favorable outcome) compared to the other IL-10 genotypes. In addition, the IL-10 (−1082, -819, -592) ACC haplotype was associated with higher DLco value compared to the other haplotypes, and the ATA haplotype was associated with a lower PaO_2_ than the other haplotypes. As such, it is possible that IL-10-mediated induction of regulatory T cells in IPF patients may play a role in the disease process. This will require further investigation.

In agreement with the reports of Riha et al. [6] and Vasakova et al. [8], we found no difference in the distribution of IL-6 alleles or genotypes between IPF patients and controls. IL-6 displays a broad range of activities, participating in the acute phase response and the stimulation and differentiation of T and B cells [26,27]. Although most nucleated cells produce IL-6, its secretion by fibroblasts stimulates the proliferation of cells in an autocrine/paracrine manner, suggesting that IL-6 is one of the key cytokines that promote fibrogenesis [28,29]. Pantelidis et al. [5] noted that the IL-6 intron 4 GG genotype was with lower levels of DLco (as a marker for disease progression) among a cohort of IPF patients from the United Kingdom. In the present study, interestingly, the GG genotype was associated with a significantly higher ground glass opacity score (i.e., active disease) compared to the CG genotype. Furthermore, the IL-6 (−174) C allele was significantly associated with lower ground glass opacity and reticulation scores versus the G allele, implying that IL-6 plays an important role in disease severity. The association between hypoxia and serum IL-6 levels in IPF patients was explored by Tsantes et al. [30], who noted that patients with profound hypoxemia (PaO_2_ < 65 mm Hg) had significantly higher serum IL-6 levels compared to healthy controls. In the present study, we found no differences in IL-6 levels between patients with PaO_2_ < 65 mm Hg or PaO_2_ > or equal to 65 mm Hg (data not shown), suggesting that mechanisms other than hypoxia are involved in IL-6 secretion in IPF patients. Moreover, we found no correlation between serum IL-6 levels and physiological parameters or CT scores in our IPF patients. However, we observed markedly elevated serum IL-6 levels in IPF patients compared to controls, implying that these patients remain in a persistent inflammatory state despite the advanced stage of fibrosis. Recently, Collard et al. [31] noted that serum IL-6 and other biomarkers of type II alveolar epithelial cells (KL-6 and SP-D) were significantly higher among IPF patients with acute exacerbation compared to stable IPF patients and those with acute lung injury. Together, the findings in the present and previous studies indicate that IL-6 may be a marker for disease progression. Future studies will be needed to explore its role in the pathogenesis of pulmonary fibrosis.

TGF-β, which is produced by a wide variety of cell types, is one of the key cytokines involved in the pathogenesis of pulmonary fibrosis. TGF-β_1_ possesses a broad spectrum of activities; it is chemotactic for fibroblasts, acts as a potent inducer of extracellular matrix synthesis, and can stimulate protease inhibitor expression [32]. Up-regulation of TGF-β gene and protein expression has been documented in lung tissues from patients with IPF and in an animal model of pulmonary fibrosis [33-35]. Furthermore, antibodies against TGF-β_1_ were found to decrease bleomycin-induced pulmonary fibrosis, further substantiating the role of this cytokine in pulmonary fibrosis [36]. In studying IPF patients of Han ethnicity, Li et al. [37] found an association between the TGF-β_1_ 869 > C polymorphism and the development of IPF. In the present study, however, we did not find that TGF-β_1_ gene polymorphisms predisposed Saudi patients to develop IPF. This is in agreement with reports from other regions, including Spanish [9], Australian [6], and Czech [8] populations. Nonetheless, the lack of association between TGF-β_1_ gene polymorphisms and the disease does not rule out the clinical importance of this cytokine in the ongoing scarring observed in IPF patients. Xaubet and colleagues [9] noted that the presence of the proline-encoding allele at codon 10 of TGF-β_1_ in IPF patients was associated with a significant increase in alveolar arterial oxygen tension difference during follow-up, compared to IPF patients without the proline-encoding allele. In the present study, the associations of the TGF-β_1_ (codons 10 and 25) TC GG, TC GC, CC GG, and CC GC genotypes with PaO_2_ and the extent of parenchymal involvement (as assessed by HRCT) suggest that TGF-β_1_ plays an important role in determining disease severity. The CC GG genotype is particularly interesting in this respect, as it was significantly associated with a higher PaO_2_ value and a lower total extent score. Relatively few patients harbored this genotype (n = 5) in the present study, making it difficult to draw a firm conclusion. Future work will be required to validate our findings in a larger sample and examine whether this genotype could be used to identify a subset of IPF patients with a more favorable prognosis.

Plasma levels of TGF-β_1_ have been shown to be under genetic control [38]. In IPF patients, the plasma levels of TGF-β_1_ were previously noted to be higher than those of controls [39,40]. However, Molina-Molina et al. [39] reported that changes in TGF-β_1_ levels did not correlate with changes in lung function parameters in IPF patients monitored over time. Here, we found no significant difference in the serum level of TGF-β_1_ between patients and controls. Also, consistent with the previous report [39], the serum levels of TGF-β_1_ did not correlate with any clinical parameter (e.g., pulmonary function tests, arterial blood gas values and CT scores) in our IPF patients.

TNF-α is a proinflammatory cytokine that plays a central role in stimulating cell-cell adhesion and transendothelial migration, as well as in the early events of the cytokine and chemokine production cascade [41]. Increased expression of TNF-α has been found in the lung tissues of humans and animal models of pulmonary fibrosis, and administration of a neutralizing anti-TNF-α antibody to an animal model was shown to attenuate pulmonary indicating that TNF-α is an important cytokine in the pathogenesis of lung fibrosis [41,42]. TNF-α polymorphisms have been significantly associated with an increased risk of IPF among Italians and Australians, but not in British and Czech populations [3,5,6,8]. In the current study, we found no difference in the distribution of TNF-α alleles and genotypes between IPF patients and controls. However, the TNF-α (−308) GG genotype was significantly associated with a higher honeycombing extent score, whereas the TNF-α (−308) AG genotype tended to be associated with a lower honeycombing extent score. This suggests that TNF-α polymorphisms may play an important role in disease severity.

IFN-γ is a Th-1 cytokine that plays pivotal roles in modulating immune responses; it can inhibit fibroblast proliferation and has been shown to reduce extracellular-matrix deposition in animal models of fibrosis [43]. Previously, IFN-γ was found to be decreased in the sera and lung tissues of IPF patients [25,44]. However, in agreement with the findings of Latsi et al. [45] and Vasakova et al. [8], we found no association between IFN-γ polymorphisms and IPF in our Saudi population.

## Conclusion

In conclusion, we herein identified associations between TNF-α, IL-6, IL-10, and TGF- β_1_ polymorphisms and PaO_2_, DLco, and HRCT scores in IPF patients, implying that these factors may play important roles in modulating disease severity. The TGF-β_1_ (codons 10 and 25) CC GG genotype, which was present in a relatively small proportion of patients compared to the controls, was associated with decreased disease severity. Further studies will be needed to evaluate the potential protective effect of this genotype against severe IPF.

## Competing interests

The authors declare that they have no competing interests.

## Authors’ contributions

EHA contributed to the design of the study and care of patients, obtained patient data, performed statistical analysis, interpreted the data and drafted the manuscript. JGC performed statistical analysis, interpreted the data and drafted the manuscript. ZC, AA, and AAA contributed to data collection and helped to draft the manuscript. All authors read and approved the final manuscript.

## Pre-publication history

The pre-publication history for this paper can be accessed here:

http://www.biomedcentral.com/1471-2350/14/66/prepub
